# The neurobiology of functional neurological disorders characterised by impaired awareness

**DOI:** 10.3389/fpsyt.2023.1122865

**Published:** 2023-03-16

**Authors:** Beatrice Annunziata Milano, Michael Moutoussis, Laura Convertino

**Affiliations:** ^1^Institute of Life Sciences, Sant'Anna School of Advanced Studies, Pisa, Italy; ^2^Faculty of Medicine and Surgery, University of Pisa, Pisa, Italy; ^3^Wellcome Centre for Human Neuroimaging, University College London, London, United Kingdom; ^4^Max Planck UCL Centre for Computational Psychiatry and Ageing Research, University College London, London, United Kingdom; ^5^National Hospital of Neurology and Neurosurgery (UCLH), London, United Kingdom; ^6^Institute of Cognitive Neuroscience, University College London, London, United Kingdom

**Keywords:** FND, Bayesian, theoretical neurobiology, predictive coding, brain modelling, resignation syndrome

## Abstract

We review the neurobiology of Functional Neurological Disorders (FND), i.e., neurological disorders not explained by currently identifiable histopathological processes, in order to focus on those characterised by impaired awareness (functionally impaired awareness disorders, FIAD), and especially, on the paradigmatic case of Resignation Syndrome (RS). We thus provide an improved more integrated theory of FIAD, able to guide both research priorities and the diagnostic formulation of FIAD. We systematically address the diverse spectrum of clinical presentations of FND with impaired awareness, and offer a new framework for understanding FIAD. We find that unraveling the historical development of neurobiological theory of FIAD is of paramount importance for its current understanding. Then, we integrate contemporary clinical material in order to contextualise the neurobiology of FIAD within social, cultural, and psychological perspectives. We thus review neuro-computational insights in FND in general, to arrive at a more coherent account of FIAD. FIAD may be based on maladaptive predictive coding, shaped by stress, attention, uncertainty, and, ultimately, neurally encoded beliefs and their updates. We also critically appraise arguments in support of and against such Bayesian models. Finally, we discuss implications of our theoretical account and provide pointers towards an improved clinical diagnostic formulation of FIAD. We suggest directions for future research towards a more unified theory on which future interventions and management strategies could be based, as effective treatments and clinical trial evidence remain limited.

## Introduction

*« The self, shelter from the storm and storm itself »: Does awareness of the self and the surrounding world protect or endanger?*

Functional Neurological Disorders (FND) have been regarded as a spectrum of clinical conditions difficult to disentangle. For over 50 years, they have been a no-man’s land between psychiatry and neurology, where even specialist physicians received limited training (1–4). Despite being debilitating conditions with a prevalence of around 50 per 100,000 people (5–8), and the second most common cause of a neurological outpatient visit after headache (9–12), FND were neglected for decades. Reasons for this include the subtle pathophysiological complexity of FND, which made theories of these disorders almost impossible to substantiate; and the historical controversy between clinical neuroscientists and psychoanalysts, which, together, put the validity of these conditions in doubt.

In recent years, however, FND have been the focus of renewed research and clinical interest (13–24).

This group of clinically heterogeneous conditions has, indeed, received descriptive diagnostic criteria in the Diagnostic and Statistical Manual of Mental Disorders (DSM-5) and International Classification of Diseases (ICD-11).

In DSM-5, diagnosis of FND requires the following criteria: one or more symptoms of altered voluntary motor or sensory function; clinical evidence of incompatibility between the symptom and recognised neurological or medical conditions; symptoms or deficits are not better explained by another medical or mental disorder (25).

However, even the latest guidelines contain ambiguities that lead to confusion between clinicians, researchers, and the public (26), making it of pivotal importance to further clarify the pathophysiology of FND. Current criteria and guidelines remain rather vague and unclear, as many symptoms of FND are not even specific to neurology, but occur within rheumatological, gastroenterological, cardiological, and pain conditions (27–29). The naming of these disorders remains confusing, still referring to specific yet controversial mechanisms (Conversion in DSM-5; Dissociation in ICD-11). Within the variety of FND, disturbances of awareness have been long recognised (30), but their neurobiology is even less understood than that of other functional symptoms, and would particularly benefit from clearer diagnostic classification.

By « Functionally impaired awareness disorders », or FIAD, we refer to the symptom dimension within FND characterising the nature and degree to which awareness is disturbed. We introduce the acronym FIAD to conceptually isolate and focus research on phenomenologically altered perception, e.g., glove anaesthesia, functional hemianopia. This is a clinical-descriptive conceptualisation, not a mechanistic one. We acknowledge but do not study, at this stage, the likely relationship of FIAD with disorders of higher awareness, such as awareness of the disorder itself (metacognition, insight). In FIAD, the individual’s ability to perceive the world and hence to respond is altered, not necessarily implying impaired cognition, or motor abilities.

FIAD encompass a range of subjectively altered awareness, ranging from focal sensory disturbance to profound global disorders such as trance states and functional coma. FND patients present with a large variety of clinical findings suggestive of disturbance of awareness. It is likely, but remains to be ascertained, that the neural underpinnings of distorted awareness *cause* distorted responsivity. The latter includes loss of reaction to sensory stimulation (including pain), loss of verbal comprehension with associated mutism (no or minimal verbal response), hypotonia, decrease or cessation of fundamental organised motor functions such as drinking and eating, and enuresis and encopresis (31, 32). Even severe FIAD such as functional coma present without laboratory abnormalities in blood and urine tests, ECG, EEG, and MRI, which are within the clinically normal range (33).

The neurobiological theory that we will delineate nuances the rigid distinction between culture-bound and functional disorders of awareness that the major classifications demand. To furnish data for this unified theory, we turn to FIAD described worldwide (31, 34), and describe Resignation Syndrome (RS) (28, 29), Traumatic Withdrawal Syndrome (TWS), Grisi Siknis (GS), and non-epileptic seizures (NES).

### Resignation syndrome

Resignation syndrome (RS), or *uppgivenhetssyndrom* in Swedish, was first described in the 1990s as a catatonic-like condition that induces a state of reduced consciousness (34). It has been compared to « culture-bound’ or « dissociative » disorders (33–35). Affected individuals (predominantly children and adolescents in the midst of a protracted migration process) first exhibit symptoms of anxiety and depression, apathy and lethargy. They then transition to a state of severe withdrawal. Eventually, their condition may become stuporous, i.e., they stop responding, eating, talking, and become incontinent. At this stage, patients are unconscious, and tube feeding is life-sustaining. This condition can persist for months, in some cases for more than a year. Remission occurs with a gradual return to normal functioning, in many cases associated with improvement of life circumstances (36).

A case series of 46 patients by von Knorring et al. (31) addressed the background of RS, highlighting the pathogenic role of trauma. In this series, all patients had experienced or witnessed violence, rape, or killings, or threats against a close family member. Almost all children (95.6%) suffered from post-traumatic stress syndrome (PTSD) and/or a depressive episode prior to resignation syndrome. Most belonged to an ethnic or religious minority (69.6%), almost all of which were persecuted (93.5%): Uighurs, Romani, Yezidis, Armenians in Russia and Ukraine. A large proportion of the children had one (28%) or both parents (30%) suffering from a mental or severe physical disorder. Remarkably, only a minority of the children came from war zones (17.4%).

In stark contrast, others proposed that RS is a behavioural disorder « induced » by families. Sallin et al. (28) argued that separating RS patients from their parents and keeping them strictly uninformed of the asylum process, would be therapeutically beneficial. This was based on a study of 13 participants in a specialist unit: 9 of them recovered, with 8 out of these 9 subjects being separated from their parents. Furthermore, the 4 subjects who did not recover within the timeframe of the study were granted asylum. However, inferences about the nature of RS here are limited by the study methodology, and especially the treatment allocation of patients to interventions. Specifically, the separated cases came from families already assessed to have problematic parental capacity. Five of these cases were committed to compulsory social care.

Resignation syndrome patients may be suffering from catatonia (34). Both conditions are characterised by a decreased ability to initiate voluntary actions, paucity of movement, stupor and mutism. RS hence fulfils three or more diagnostic criteria for catatonia (25). DSM5 and ICD11 describe catatonia as a manifestation of another diagnosis, not a primary primary one, begging the question of whether in RS it is a manifestation of (complex) PTSD. Unlike in catatonia, increased limb tone, echolalia, echopraxia, and mannerisms are not found in RS, which is typically much longer lasting than catatonia. RS appears to respond poorly to benzodiazepine treatment (37).

### Traumatic withdrawal syndrome

Traumatic withdrawal syndrome (TWS) has been observed in refugee children transferred from Australia to the Nauru Regional Processing Centre in the past decade (38–41). Since 2012, 222 children, of whom at least 27 were unaccompanied, have been sent to Nauru (42). These asylum seekers were mainly from Iran, Pakistan, Sri Lanka, Pakistan, Bangladesh, low-income African countries, or stateless (43). The clinical features included pervasive social withdrawal, severe reduction or inability to walk, talk, eat, drink, self-care, and socialise. Children would actively resist or not respond to acts of care and encouragement (44). TWS was mostly documented in females aged 7–15 years, but was also found in adult males (45).

### Grisi siknis

Grisi siknis (GS), prevalent amongst the Miskito people of eastern Central America, primarily affects young women between the ages of 15 and 18. It was first described in detail by the anthropologist Philip Dennis after he had come across it in the 1970 (35). Symptoms appear to be anticipated by anxiety and headaches, culminating in long periods of coma-like unconsciousness, with sudden outbreaks of violent and aggressive behaviour. Many cases were associated with gender-based violence and oppression, as the young women were pressurised into sexual relationships with older men (46). It may be argued that the symptomatology associated with the condition may avert this harassment (35). Yet, little is known about how exactly the clinical and gender dimensions of GS relate to the socio-political context in the region.

### Non-epileptic seizure disorder

Non-epileptic seizure disorder (NES) remains one of the most common presentations of FND in industrialised countries (47). NES is a spectrum of paroxysmal behaviours that resemble epileptic seizures, but lacks macroscopic abnormal electrophysiological activity. They involve impairment of consciousness, flaccid or rigid collapse, and/or tremulous limb movements (48). Patients give rich and varied accounts of disturbance of awareness during seizures. NES are clinically challenging to diagnose (46); the gold standard is Video Electroencephalogram (vEEG) monitoring, where the events are monitored by continuous video recording and simultaneously co-registered with Electroencephalogram (EEG). When a patient’s habitual event is captured on vEEG and the clinicians are provided with a complete patient history, diagnosis of NES can be made with high confidence (47–64).

## Neurobiological theories: A historical perspective

We find a concise historical understanding of the neurobiology of FIAD to be essential to inform future development and directions in scientific research, clinical taxonomy, diagnosis and treatment.

The first attempts to integrate neuro-psychological understanding of FIAD took place in the late 19th century; yet, from the 1920s until the first decade of the new millennium, neurobiological aspects of FND and FIAD were broadly disregarded by clinicians and researchers. As a result, FIAD missed out on the revolution in biological – and indeed biopsychosocial - understanding, still standing out at the intersection between neurology and psychiatry (65, 66).

In particular, Charcot, Janet, Breuer and Freud were the main contributors to the development of theories of FIAD in the late 19th century (67–69). Charcot worked with FIAD under the rubric of « hysteria »^1^ demonstrating cases of apparent loss of consciousness and vigorously rejecting simplistic gender-based theories and treatments (70–79).

Janet’s enduring legacy to the neurobiology of FIAD was his dissociation theory (80). The latter theorised that neurobiological vulnerability, especially in traumatised patients, led to a fragmentation of psychological functions under stress, which he saw as a lesion rather than a defence. He proposed attention to play a crucial role in the pathophysiology of FIAD, and that its withdrawal was to be held responsible for the onset of symptoms (81). Janet saw functional sensory loss as a key symptom and hypothesised an abnormally high level of activity in the mechanism normally filtering out extraneous sensory input, well accounting for loss of function (64). Moreover, he maintained distraction to be an alleviating solution for functional tremor (80–82). The concept of dissociation has developed since Janet, and is a major heading in ICD-11 (60–66), where it describes a discontinuity in the integration of brain functions as central to FND.

In the famous « Studies on Hysteria » (1895), Breuer and Freud developed Janet’s dissociation, or splitting, of mental functions (83–87). Here, excessive excitation caused by emotional events would be « converted » into somatic phenomena, leading to hysteria (88, 89). Crucially, Freud greatly emphasised the defensive function of this « conversion », which later formed the only aetiologically based category in DSM-III, and bequeathed the description of Conversion Disorder » (CD) in DSM-5. In a similar vein, « La belle indifference », defined in DSM-5 as a lack of concern about the nature or implications of the symptom » (25), is listed as a feature supporting a diagnosis of CD. The neurobiological claims of the dissociative and conversion traditions, however, give a rather *ad hoc* account of why many signs of FND require attention to manifest (i.e., paralysis, tremor) and why some may improve when attention is diverted (64).

Kretschmer (90, 91) described two behavioural responses to threat observed in animals, comparing these instinctive patterns to FIAD symptom patterns, such as convulsive dissociative seizures and violent tremors, and paralysis and dissociative seizures. He was also the first to hypothesise that behaviour triggered initially by a traumatic event or by a stress-induced response, through repetition, becomes increasingly habitual and automatic: a conditioned response that no longer required the presence of the inciting event (91).

With the later decline of psychoanalytic psychiatry, interest in FIAD and « hysteria » waned and its « near total disappearance » as a diagnosis was announced (92). In 1965, eminent British neurologist and psychiatrist Eliot Slater went further, maintaining that hysteria had never existed, but rather was the result of misdiagnosis (92–95). In the 1980s and 1990s, most doctors disregarded these patients, who had become « almost literally invisible to medicine, and modern medicine’s untouchables» (95, 96).

Still some progress was made. In 1967, Whitlock (97) proposed a biological hypothesis for FIAD. Inhibition of afferent input at the level of the reticular formation could result in « selective depression of awareness of a bodily function » (25). Hence, attentional diversion away from the symptomatic region inhibited afferent input from that region, resulting in loss of function (98–119).

The end of the 20th century and the start of the 21st (120–159) saw a revival in the clinical and scientific interest in FIAD, due to advances in clinical neurology research and recognition that large numbers of patients were unfairly blamed and stigmatised for their disabilities.

It was indeed in this context that CD gained recognition in the clinical setting, emphasising the presence of neurological symptoms of a physical ailment with no corresponding organic cause. CD would, therefore, suggest that psychological suffering and tensions could be « converted » into physical symptoms, such as paralysis, blindness, loss of speech and/or seizures (111, 120, 136).

Overall, a growing number of neuroscience studies has been conducted since, starting to demonstrate the anatomical and functional circuitry of FND. However, our understanding is limited, and mostly about motor symptoms rather than FIAD.

Therefore, we aim to draw on recent neuro-computational principles and the attention-focused work of Edwards et al. (64), to provide a neurobiological account of FIAD integrating the « lesion » (dissociative, deficit) and « purposive » (conversion, defensive) traditions. Within a Bayesian, « active inference » framework, we propose that FIAD may be caused by maladaptive neurally encoded beliefs about the state of the world and the optimally attainable conditions to live in it. Thus, acknowledging the variety of predisposing (i.e., psychosocial adversity, gender, physical illness, exposure to symptom/illness models), precipitating (i.e., physical injury, mental health symptoms, interpersonal conflict, other stressors) and perpetuating (i.e., avoidance, illness beliefs/expectations, social isolation) factors. Finally, we lay out potential clinical implications of this account.

Better models can further inform clinicians about the genesis and the clinical trajectories of these conditions and strengthen collaborative treatments between families, patients, and professionals.

To this end, we highlight directions for future inquiry likely to yield high impact advances.

## Results

We now review the current state of knowledge about FIAD, based on a comprehensive literature search. This included reviewing clinical reports, neuroimaging studies, theoretical, and computational neuroscience accounts. Details of the methods supporting this review of the literature are found in the Methods section below.

### Contemporary neuroscience of functional neurological disorders with impaired awareness

More recent studies illustrate the likely neurobiological and pathophysiological pathways that may underpin the impaired awareness process in FND. They do not, however, clearly explain how a psychological stressor might be related with a particular sign or symptom, such as loss of response to sensory stimulus, loss of comprehension and social contact, or disturbance of fundamental motor activities (48, 49). Growing evidence suggests that psychological stressors (in childhood and adulthood, and whether remembered or not) are linked to biological changes (50, 51) as measured by stress biomarkers (cortisol, amylase, heart rate variability, brain activity, and epigenetic changes) (52, 53) and measurably altered neural activity may be associated with FIAD.

Specifically, the literature suggests that mechanisms underpinning attention, emotional processing and interoception could contribute to the altered perception of internal and external states (160–179). Computational models explaining how the embodied brain responds to psychosocial stressors can be reconciled with recent neuroscience evidence on the above-mentioned mechanisms (180–183).

Yet, the reader should notice that focusing on one mechanism at the expense of the others might result in a reductionist description of isolated components, with little regard for the complexity beyond the sum of its parts. Specifically, it is unlikely that a single direct relationship between stressful or traumatic events and FND would provide a sufficient explanation. Rather, the aetiology is more likely to be complex and entail a variety of predisposing, triggering, and perpetuating elements (184–189).

In presenting the available neuroscientific evidence on possible mechanisms of FIAD, it is important to underline how, whilst the literature on FND with motor symptoms is rich, there is little available experimental evidence to account for a mechanistic hypothesis of FIAD. Here, we summarise the available evidence and connect it with theoretical work, with the aim to inform much needed development of further scientific investigations.

Altered awareness is commonly found in healthy people during highly stressful situations, and it is probably an adaptive response, such as analgesia to major injuries in battle (189–196). Clinically, intense emotion, stress and trauma have often been associated with degrees of loss of awareness, but theories regarding their causal roles remain highly controversial (197–201). Recent progress has led towards a more nuanced knowledge of emotion in FND, which move beyond a simple dichotomy between stress-induced or not. Numerous interdependent emotion processing activities are currently thought to be mapping onto salience and other limbic/paralimbic (e.g., ventromedial and orbitofrontal prefrontal cortex, parahippocampus, hippocampus, dorsolateral prefrontal cortex) circuits (202).

The « salience network » appears to recognise and respond to one’s homeostatic demands; it consists of the dorsal anterior cingulate cortex, anterior insula, dorsal amygdala, periaqueductal grey (PAG), and hypothalamus (203–208). Enhanced amygdala and PAG bottom-up activation appears to be related with increased emotional reactivity, arousal, and protective reactions. Patients with FND had decreased amygdala habituation and higher sensitivity during processing of negative emotions. Alterations in the control of amygdala and PAG activation by the prefrontal cortex may also contribute to heightened emotional reactions (209).

Individuals with mixed active FIAD symptoms had higher baseline arousal levels than those with anxiety disorders or healthy volunteers, as measured by spontaneous changes in skin resistance and failure to achieve acclimation to repeated auditory stimulation and sonic stimulation (210–223).

Yet, studies often show seemingly contradictory results. Bakvis et al. (224) reported that 19 NES patients had elevated basal diurnal cortisol levels and lower heart rate variability at baseline, indicating increased sympathetic activity (225, 226). Van der Kruijs et al. (227), instead, when employing positive outdoor images and a Stroop test found no activation differences between NES patients and healthy controls. However, stronger connectivity values between areas involved in emotion (insula), executive control (inferior frontal gyrus and parietal cortex) and movement (precentral sulcus), significantly associated with dissociation scores were retrieved in NES patients (120, 227–234).

Moreover, in a single within-subject fMRI case study on CD, using a vocalisation task, Bryant and Das (235) found that inferior frontal gyrus activity was positively functionally connected to anterior cingulate and negatively to amygdala activity during speech recovery, but not during mutism. Therefore, suggesting a link between speech networks and the anterior cingulate, which controls amygdala activity (236–238).

In a functional paralysis case study, Kanaan et al. (239) found increased amygdala and right inferior frontal activity, as well as decreased motor activity.

Aybek et al. (240) evaluated 12 CD patients’ responses to sad or scary vs. neutral faces (in comparison to controls matched for age, gender, IQ, and sexual trauma). Left amygdala, premotor/sensorimotor area, cingulate cortex, and PAG showed higher activity. The authors would, therefore, propose that cumulative fear sensitization in the amygdala may cause long-lasting physical reactions to stress and danger.

As PAG activation mirrors animal « freeze reactions » to scary stimuli, a potential biomarker for FND may be the absence of fear conditioning in the amygdala (120, 241–245).

### Everyone knows what attention is and no one knows what attention is

Over the course of time and across different disciplines, attention has been addressed and presented with many definitions (246, 247). Interestingly, it has been defined as the process that enhances representation of some kinds of information and inhibits others (248), thus privileging the former over the latter for further processing. It has long been hypothesised that « dissociative » disorders, and FIAD, in particular, involve biased attention, we researched the sources that cited original search articles to find (249–255). For example, experimental participants scoring high for dissociation on a self-report scale showed reduced attention to somatosensory stimulation after they watched a trauma-related film (162, 256–260). As we will describe below, predictive coding theory formulates attention as an implicit prediction about the value of sensory input. In other words, patients may gate out awareness of input, analogous to collateral discharges normally suppressing (predictably uninformative) blurring during visual saccades. Thus, predictive coding theory both emphasises and helps operationalise the role of attention in FIAD.

**Figure 1:**
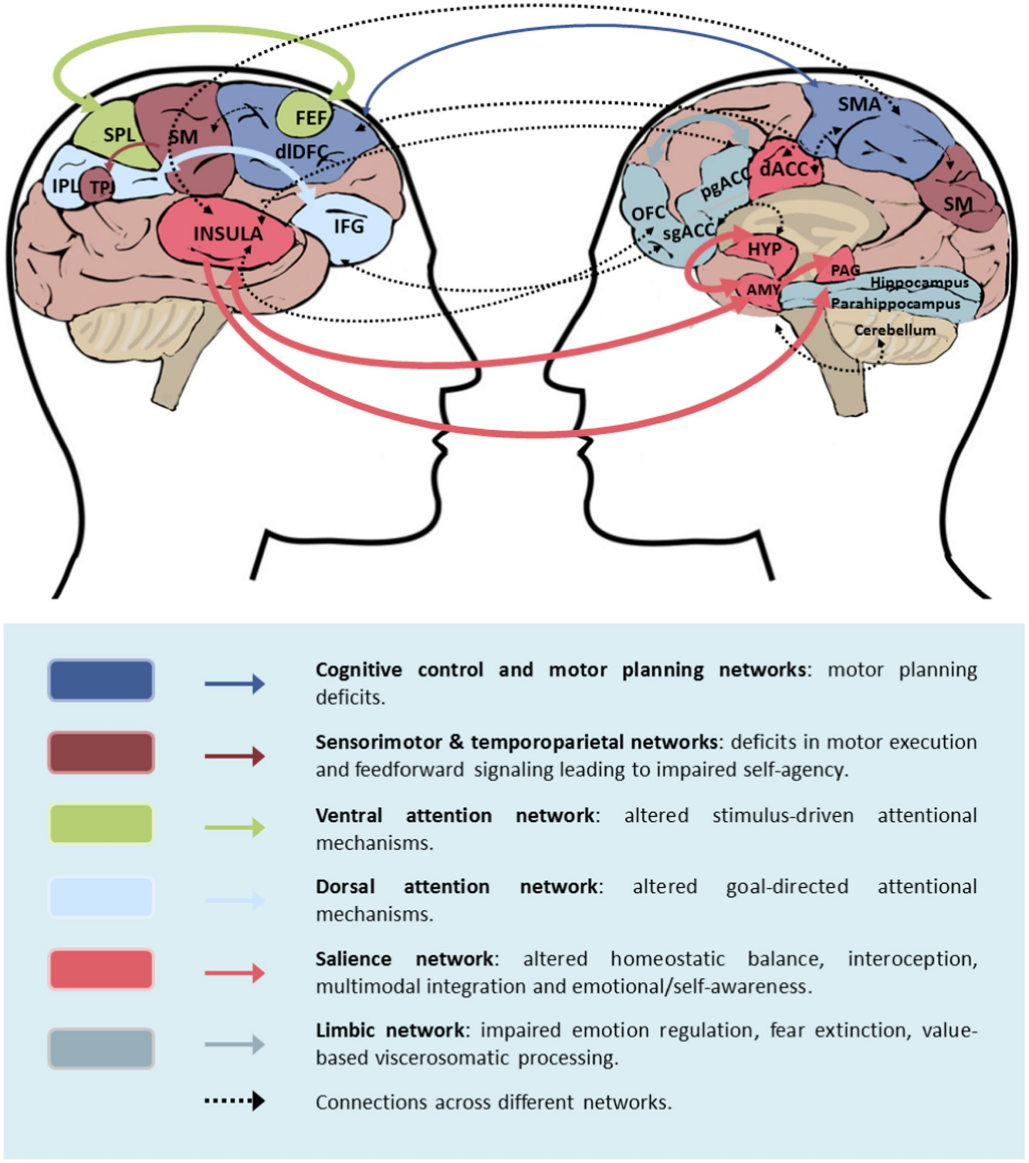
Neural correlates of FND involving impairment of awareness (FIAD) FIAD may include altered connectivity within and across brain circuit. This figure, adapted from Drane et al. (209), shows with thicker arrows the connections more likely to be involved in FIAD, amongst those implicated in FND in general. AMY, amygdala; SMA, supplementary motor area; dACC, dorsal anterior cingulate cortex; dlPFC, dorsolateral prefrontal cortex; HYP, hypothalamus; OFC, orbitofrontal cortex; pgACC, perigenual anterior cingulate cortex; sgACC, subgenual anterior cingulate cortex; SM, sensorimotor area; TPJ, temporoparietal junction.

Neural correlates of attention characterised by disruptions in sustained and selective attention have been focus of investigation (Figure 1).

Kowloska et al. (261) demonstrated in this study that children and adolescents with acute conversion symptoms have a diminished capacity for knowledge manipulation and retention. Upon completion of the IntegNeuro neurocognitive battery by 57 participants and their matched healthy controls, FIAD patients appeared to have a diminished capacity to block and interfere with information and to inhibit responses, all of which are required for effective attention, executive function, and memory.

In this prospective case–control study by Stager et al. (262), 26 children with video-EEG-confirmed non-epileptic seizures (NES) performed a modified Stroop test. Here, NES patients showed poorer selective attention, less awareness and greater cognitive inhibition than controls.

Interestingly, the core regions of interests identified found in neuroimaging studies of FIAD are part of the attentional network and have been hypothesised to play pivotal roles in processing prediction and modulating multiple levels of the brain hierarchy.

In particular, the anterior cingulate cortex and the posterior parietal cortex have been related to a regulatory role in modulating the weighting of long-term goals, encoded by the prefrontal cortex (PFC) vs. short-term goals, encoded by lower-level areas.

The inhibition of short-term goals and prioritisation of long-term planning is also associated with the activity of interior frontal gyrus (IFG) and ventrolateral prefrontal cortex (vlPFC), associated with top-down inhibition of the amygdala and increased connectivity between IFG and vmPFC (209).

Complementary scientific evidence therefore suggested that the inferior frontal cortex identifies conflicts in perceptual cues (sensory information) and conscious experience and translates ambiguous sensory input into precise conscious experience.

Recent evidence found the periaqueductal grey (PAG) not only involved in autonomic control, but also engaged in mechanism of cognitive control and in encoding the probability of threat, rather than fear output *per se* (209).

Although not directly studied in FIAD, it is worth noting that an interoceptive processes have been investigated in the context of FND and could potentially be of interest in the study of FIAD. Interoception is a bidirectional process characterised by feedback and feedforward loops that map bodily states (263–268).

In the next section, we will introduce foundational concepts of the Bayesian brain hypothesis and the active inference framework, reconnecting the neurobiological plausible hypotheses to current neuroscientific evidence. We will finally show how this theoretical framework can inform future research and hypothesis testing.

## FIAD and the Bayesian brain

We now turn to neural predictive coding, and specifically *active inference* (AI), as a useful framework to understand FIAD. It can help formulate rigorous neurobiological hypotheses, describing how changes in neural activity and connectivity could result in modulation of motor and sensory gating leading to FIAD.

### Active inference processes relevant to awareness

Active inference (269–275) posits that all organisms hold an implicit model of « how life should unfold », in terms of both homeostasis and development. Deviations, or expected deviations, from this scheme then motivate corrective processes, such as learning, moving or eating. Thus, active inference can be thought as a normative framework. The actions that *I believe* that I will take are the ones consistent with a *belief that the best outcomes will be obtained.* Deviations from (adaptive) predictions are *surprising,* so that organisms must minimise this type of surprise, furnished by their sensory information, through controlling their actions to *resolve uncertainty* towards belief in benign observations to come (Bayes-optimal behaviour). Technically, « how life should unfold » is called the generative model of the world and the self. Hence, the brain is fundamentally a predictive machine, performing probabilistic computations to infer the causes of sensory evidence and the likely outcomes of actions (276–282).

Computationally, living beings are thought to reduce the surprise inherent in « how life is unfolding », relative to « how it should unfold », by dynamically comparing their model of the world (prior expectations, or predictions) and their observations (sensory input) (64, 269, 283) in terms of a measure of surprise called « free energy ». The comparison between model expectations and observations results in prediction error; in biological terms, the minimization of the prediction error (or surprise) is achieved by modulating synaptic activity and neuronal connections. Action and perceptual inference both contribute to free energy minimization, the former by sampling new salient sensory evidence to reduce uncertainty, the latter by updating the initial expectations (Bayesian belief update) of the agent to better account for observations (64).

Our expectations crucially depend on the true states of the world which cause our sensory observations, true states which are hidden from us and are thus only represented in the generative model as probabilistic beliefs. Their update demands probabilistic, or Bayesian, inference, which is not always objectively accurate. This is because of discrepancies between the person’s generative model, which may not correspond to the true process generating their observations, and which we postulate is important in FIAD; and to the limited resources of the human brain resulting in approximate inference.

Importantly, the communication of information within the brain is hierarchical. According to predictive coding theory, each level of the hierarchy informs the level below with expectations regarding observations, based on their most likely (hidden) causes. Different neuronal populations are responsible for encoding predictions (prediction units) and prediction error (prediction error units). Prediction errors are estimated at the lower levels and propagated to the level above, where they are used to adjust beliefs, hence minimising free energy at each consecutive level. This hierarchical structure naturally implies that higher levels encode states operating over longer time-scales than lower levels, which will become important when we reconsider the computational neuroanatomy of FIAD below.

Central to active inference is the concept of precision, which is very useful to understand FIAD. Precision, or inverse variance, can be interpreted as the confidence of the model in its prior beliefs and sensory observations, which determines their relative contributions to the updated beliefs resulting from the comparison between (top-down) expectation and (bottom-up) sensory evidence. Highly precise (prior) beliefs, or very uncertain sensory evidence, will require the brain to sample more evidence to shift its beliefs. On the other hand, great confidence in the sensory evidence (or very imprecise prior beliefs) would result in a substantial update of expectations even in the presence of sparce new evidence. In this case, the prediction error would be mostly driven by sensory information (284). It has been proposed that superficial cortical pyramidal cells encode the precision of the prediction error *via* synaptic gain.

## Discussion

Functional disorders of awareness lacking a unifying theoretical framework, resulting in fragmented clinical understanding and management guidelines. A unifying theory of FIAD, based their functional neuroanatomy reviewed above, would be beneficial for clinicians and patients alike. Moreover, the symptomatology associated with the condition poses challenging theoretical questions in neuroscience that, if answered, would increase our understanding of the basic physiological mechanisms of awareness and elucidate its pathology (284–287).

### Actively inferring to be un-aware

Building on the work of Edwards et al. (64), we hypothesise that FIAD are generated by internal *models of disease compatible with the clinical presentation, often shaped by adversity, which guide the gating of attention and hence, of awareness.* In the case of RS, the adversity in question is severe and concerns both the index patient and their family. The *models of disease* consist of illness-compatible prior beliefs, which in the ill state are endowed with excessive precision by attention. Aberrant attentional processes can be caused by learning-based alterations of synaptic connectivity and/or by a primary predisposition or « preparedness » to attentional biases. In what follows, we describe how Bayesian brain theory, and specifically the active inference framework (AI), maps this attentional theory into a biologically plausible hypothesis explaining FIAD. This is likely to be helpful to direct future research, is in principle testable, and helps to de-stigmatise these conditions.

From the point of view of AI, any illness consists of inferring a state of harm (e.g., a bacterial infection) and the optimal actions (e.g., inflammation), on the basis of a generative model of the world, as we saw. This model is equipped with different types of prior beliefs (priors) which play complementary roles in modulating behaviour. Aberrant priors and their modulation can result in « unwarranted symptoms ». A key example of such priors are the beliefs over hidden states, which will cause sensory observations (the state-to-observation map is the « likelihood matrix » in AI). These are particularly relevant in neuropsychiatric conditions, for example « I experience palpitations (observation) hence a heart attack is likely (prior in panic disorder) ».

The patient’s model of themselves may contribute to symptomatology. If the purpose of the generative model is to explain the hidden (not obvious) origins of sensory input, then the patient’s own perceptions must have their underlying cause inferred. In certain situations, a state of ongoing suffering could be best explained by inferring that « I » (as the suffering entity) « am in a patient » (as in a sick) « state ». In the case of RS, « I do not experience pain » would include both « this pinprick is not disturbing » and « my sickness disturbs nociception » as components of the patient’s family’s generative model. To eliminate prediction errors caused by the expectation « I am sick » and the sensory evidence of my biological processes, the patient’s behaviour would conform to the concept of disease. This self-fulfilling prophecy would normally mediate recovery (e.g., « exhausted people rest »), but here may explain the progression of the symptoms, which are often outside conscious control.

Within the paradigm of AI, modulation of the strength of the likelihood matrix compared to the priors weighs the impact of one’s observations, and is hence regarded as *attention control*. Consequently, different symptomatic behaviours might emerge as Bayesian optimal for a model with aberrant priors depending on their domain and their modulation, positive or negative modulation, making them stronger or weaker. In the case of FIAD, both psychological and physical events may play important precipitating roles in predisposed individuals, namely those whose model of the world is particularly prone to extreme modulation of precision. Here, the brain’s hierarchical organisation, in which prediction at higher levels (empirical priors) infers both current happenings and most proper answers at lower levels, is crucial. If exposure to inescapable trauma, for instance, has strengthened the connection between interoceptive correlates of threat and a very precise prior belief that sensory evidence is useless (uninformative - what to do), this would make it optimal for the patient to reduce his or her sensory precision, and consequently level of awareness of sensory evidence.

Suppose, for instance, a patient with RS is endowed with highly precise prior beliefs that his current status prevents the occurrence of suffering, which would follow from responding to new information. Hypothetically, the precision of such beliefs could be learnt by an excess of aversive evidence. As common (yet anecdotal) example, RS sufferers are often the member of the family that has translated for their family in their distressing dealings with immigration authorities. Such learning could establish a precise expectation of the world’s state, and a lack of confidence (i.e., precision) over the ability of fresh sensory input to give contradictory evidence. In this setting, even random events may be classed as confirmatory signals, which would maintain inaccurate beliefs by bolstering earlier expectations. Thus, basic symptoms of RS, including weakness, social withdrawal, and cognitive disengagement, may reflect very accurate prior beliefs regarding the (lack of) usefulness of any action in reaching a desirable end. In other words, the cost of any action exceeds its utility if it does not contribute to the resolution of uncertainty (*cf.* « learned helplessness ») (269). In computational terms, it is possible that this process could not only involve aberrant priors’ modulation over likelihood matrix (mapping between hidden states and sensory evidence), but also over policies (actions) and transition matrix (transition between states).

At the present state of knowledge, different hypotheses, involving different « pathological » model parameters, could potentially provide different causative explanations for similar symptomatologies. The power of a computational approach to the study of FIAD is the ability to clearly formulate and explore such hypotheses with in-silico models of the disorder, which can be consequently tested with experimental paradigms. Model fitting and Bayesian model comparison are powerful tools to test contrasting hypotheses and identify the level of the hierarchy and the neuronal populations (encoding different priors and prediction errors) most likely responsible for different conditions.

### Possible brain substrates for neural computations in FIAD

Reconnecting this theoretical framework to experimental evidence on FIAD, we note that one of the functions of the attentional network is to implement cognitive control over long-term (cognitively complex) goals, which we can now associate with higher precision of prediction from PFC, over immediate (less cognitive demanding) ones. The aforementioned anterior cingulate cortex and posterior parietal cortex are hypothesised to modulate the relative precision of prefrontal cortex (PFC) at the higher level of the brain hierarchy, as well as of lower-level areas. Once PFC top-down predictions are established, long-term goals are implemented by contextualising or downregulating lower-level short-term goals.

The increased connectivity between vmPFC and top-down inhibition of the amygdala, important for prioritising long-term planning (209), would also speak to top-down control, where stronger predictions from the higher levels of the hierarchy, working at longer temporal scales, would over-rule lower-level predictions, associated on faster time scales. This implies that long-term policies (associated with long-term gain or optimal behaviours), would result in maladaptive « here and now » symptoms.

In an unpredictable context, where the prediction error from sensory input cannot be easily reconciliated with one’s priors (in repeated trauma, unforeseen circumstances, lack of evidence that one’s actions lead to positive change etc), the possible conflict resolution role of the IFC might become predominant. We might also speculate that, in the presence of precise higher-level priors and imprecise (conflictual) sensory evidence, the IFC might bias posterior beliefs, and hence conscious experience, towards the upregulated priors.

Moreover, in active inference terms, the « probability of threat », encoded by the PAG, would represent the prior expectation over the hidden state « threat » causing sensory evidence. This is plausible especially once we consider the typical persistence of FIAD symptoms even when the contingent threat is not present anymore. Highly precise priors can guide behaviour even in the absence of the supporting sensory evidence, whilst a precise (prior) expectation of threat requires more contradictory sensory evidence, over a long time, to be updated from high to low probability of threat.

Finally, it is likely that interoceptive biases lead to altered awareness. As we saw, inappropriate interoceptive attention may disproportionately alter the weighting of top-down or bottom-up information streams, resulting in abnormally amplified or reduced sensory perceptions (i.e., diminished visual, auditory, skin sensitivity, or impaired consciousness) (263, 274). Within a predictive coding explanation of interoception, sensory regions transmit ascending prediction errors, which are compared with descending predictions across a hierarchy of perceptual processing (284). Interoception has been extensively discussed as a component of the predictive brain that sustains homeostasis (197). The bottom-up interoceptive prediction error would pass information about the need for actions (such as « eat ») to maintain the body homeostatic needs. In patients with RS, loss of weight and lack of active food intake, would suggest the presence of depressive modulation of the interoceptive bottom-up prediction error, overtaken by higher-level precise predictions.

## Clinical implications for the diagnostic formulation of FIAD

A computational neuroscience perspective on FIAD has important preliminary implications for the clinical understanding, diagnostic formulation, and planning optimal data collection to ensure future progress. Many functional neurological conditions are diagnosed and treated relatively successfully, but this cannot be said for the more severe conditions, especially RS (28, 240). Explicitly hypothesising that RS is a form of severe FND goes further than seeing it a culture-bound syndrome, which easily underplays its neurobiological and clinical significance, and further than purely somatic characterizations such as « state », which can underplay its psychology and sociology.

First, it is imperative for the phenomenology of RS and related FIAD to be recorded as meticulously as the somatic features have been. Fragmentary accounts indicate that subjective experiences leading into and out of RS are comparable with those of other severe dissociative states, such as derealisation-depersonalisation and NES. This includes selective abolishment of aspects of awareness, and relative preservation of others, esp. auditory, dream-like states and subjective feelings of safety-seeking.

Second, it is well recognised that there are multiple paths to each functional neurological disorder, with specific predisposing factors being statistically important but by no means universal or diagnostic, so no single pathway needs to be identified. As a key example, childhood and recent trauma is a well-recognised risk factor, but it is neither necessary or sufficient (162). Health professionals treating RS and related conditions need to take trauma very seriously, without assuming that it is necessary. If substantial trauma is present, the presence of RS would signify a great vulnerability of the individual to further traumatization.

Third, a computational perspective is highly consistent with *models of illness* structuring FND, and particularly severe FIAD like RS. In pain and depression research, it is well established that expectations (i.e., placebo antidepressant or open-label analgesic placebo; placebo antidepressant) recruit complex neural systems substantially overlapping with the respective biological interventions. These « models of illness » may be partly innate, similar to « panic without panic » in NES, and partly learnt from the subculture and from personal experience (*cf.* NES in people with a family or personal history of epilepsy). It is common for severe FND to worsen with time over months and years, thanks to the vicious cycles of inference described above (*cf* deconditioning theory).

We propose that RS is a variant of *Trance disorder* of DSM-5 and ICD-11, in that it is also characterised by « a marked alteration in the individual’s state of consciousness or a loss of the individuals’ customary sense of personal identity in which the individual experiences a narrowing of awareness of immediate surroundings or unusually narrow and selective focusing on environmental stimuli ». This dovetails perfectly with the central role of the regulation of attention that the « models of illness » above entail. However, RS would be differentiated by the absence of characteristic motor patterns found in Trance disorders. Yet, a neurocomputational view of FIAD would argue against trying to precisely separate such clinical syndromes from each other, as the learnt component of FND is certain to vary the presentation. For example, our view would relativise the distinction between culturally adaptive vs. maladaptive FIAD, as the major classifications now attempt to do for trance and related disorders.

RS would then be a confluence of the *dissociative, innate defensive and autonomic/interoceptive activation* factors listed by Koslowska et al. (29) and summarised in Table 1 below. A key point is the overlap between the models proposed by these workers, whilst these categories provide structure for clinically assessing patients with suspected severe FIAD. Of course, serious cerebral malfunction as found in minimally conscious or persistent vegetative states due to macroscopic brain insults need to be excluded. Social, psychological, biological factors, and comorbidities should be documented and a biopsychosocial framework adopted since pathophysiology occurs in a body that cannot be considered an isolated system, as we have seen regarding the acquisition of prior beliefs. The five clinical models described by Koslowska et al. (29) constitute hypotheses for biological components of this biopsychosocial approach. Detailed clinical documentation and research need to map their features in severe FIAD, and pharmacotherapeutic trials may help distinguish dissociative from catatonia-like patients, when a difference is indeed found. Prefrontal cortical function should be decreased in the latter and increased in the former, with altered neurotransmitters of the opioid and GABA systems, respectively.

**Table 1:** Five neurobiologically informed clinical models following the work of Kozlowska et al. (29).

Neurobiologically informed models for clinical purposes	Signs and symptoms	Suggested pathophysiology	Some clinical examples
Model 1: Sustained autonomic hyperarousal	Numbness, apathy, perplexity, withdrawal, fatigue, or exhaustion (18). Normal heart rate and blood pressure alternate with events of tachycardia and hypertension. Panic attacks, hyperventilation, flashbacks, nightmares, vomiting, enuresis, encopresis, and profuse sweating may be present	Proposed model in the context of polyvagal theory. Autonomic arousal due to sympathetic and defensive parasympathetic (vagal) activities	Otasowie et al. (249)
Model 2: The innate defense model	Autonomic symptoms: loss of motor function and changes in sensory and pain-processing; activation of innate shutdown responses of tonics immobility, collapsed immobility, or quiescent immobility	Two key components: the autonomic component involves a relative withdrawal of sympathetic activity and coactivation of the defensive parasympathetic component (vagus); the motor sensory component involves activation of the *ventrolateral PAG,* which mediates immobility	Von Knorring et al. (31)
Model 3: Catatonia	Agitation, catalepsy, echolalia, echopraxia, grimacing, mannerisms, mutism, negativism, posturing, stereotypes, stupor, waxy flexibility	Decreased function in the *prefrontal cortex*, with perturbed central *GABA metabolism*. Patients are more likely to respond to benzodiazepines and electroconvulsive therapy	Dhossche et al. (250)
Model 4: Hypometabolic state model	Hibernation-like state, torpor	Cells of the body enter a cell-danger response, a state in which mitochondria decrease metabolism to enable the organisms to survive a hostile environment	Naviaux et al. (251)
Model 5: The defence cascade model of dissociation	Discontinuity in the normal integration of consciousness, memory, identity, emotion, perception, body representation, motor control, and behaviour	Increased activation in the *prefrontal cortex* deactivates the *amygdala*. Perturbed *mu* and *kappa opioid systems* across the neural network and down-regulation of the sympathetic nervous system *via* the *ventrolateral PAG*. Patients are more likely to respond to opioid receptor antagonists	Schauer et al. (252)

Clinically, the proposed Bayesian-brain framework can also form the basis for a collaborative, psychoeducational model of illness that can be used to help families with members with severe FIAD.

This is because first, it makes it easy to accept and validate these conditions as a real, severe and brain-based, whilst at the same time encouraging agency. Second, and drawing from the best of FND care, it may replace an approach whereby clinicians seek to manipulate patients back into health, and patients’ families attempt to manage clinicians, with a truly collaborative one. Third, such collaborative care means that the family (or more generally, the social system) provides the patient with an environment within which they can actively infer their actions.

## Recommendations for future research

This review aimed to provide a more coherent framework for FIAD, and Resignation Syndrome in particular. Overall, we found that research evidence in the field is still at its early stage. Whilst FND with motor symptoms have received increasing scientific interest over the last two decades, experimental research on FIAD is limited. A closer look on the available results found some evidence for the involvement of the emotional processing, attention and interoception networks in FIAD, with some conflicting results. Such conflicts may be the result of small sample numbers, variations in FIAD subtypes, methodological or task discrepancies, and indeed lack of international guidelines in identifying different conditions with overlapping symptoms. Further research is needed to advance the field with reproducible evidence. Further studies may focus on specific signs and symptoms of lack of awareness or responsivity, with attention to the differences between acute and chronic symptoms, as well as between current and remitted symptoms. Both basic neuroscience and clinical research play a fundamental role in advancing our understanding of FIAD.

Crucially, we propose a conceptual model of how the active inference framework may be used to study these conditions. It is of particular interest how different areas and networks found to play a role in FIAD naturally fit with predictive coding hypotheses, which can be computationally modelled and simulated, and finally experimentally tested. Aspects such as the role of learning *via* stress and trauma, attention modulation, functional and effective connectivity, and the computational role of different levels of brain hierarchy need to be further investigated. In future accounts, we envisage that the family-social system can be included meaningfully in such simulations, providing insight into the inter-personal development, persistence and purposes of shared models of disorder.

Understanding the similarities and variations between various presentation of FIAD may lead to the development of alternative treatments, management and prevention.

## Concluding remarks

Although the last decade has seen an increase of material on the motor symptoms of FND, a similar systematic approach to the impaired awareness symptoms of FND is sparse. We have proposed, however, that computational approaches to the study of the brain, and Bayesian hierarchical accounts in particular, could be beneficial to inform, conceptualise and guide future research on FIAD. Consistently with previous Bayesian accounts of FND and with the available experimental evidence on FIAD, we proposed that attentional processes and top-down control mechanisms might be particularly important for the aetiopathogenesis and evolution of symptoms in FIAD. Additionally, we have maintained that psychological trauma and social stressors are important factor, although not always necessarily required to induce FIAD symptoms. In clinical practice, increasing scientific interest, data availability and structured assessments throughout the diagnostic and treatment process would be fundamental to offer clinicians a solid understanding of the patients’ symptomatology, based on individual models of the world. This could guide the development of customise treatments.

## Methods

**Figure 2:**
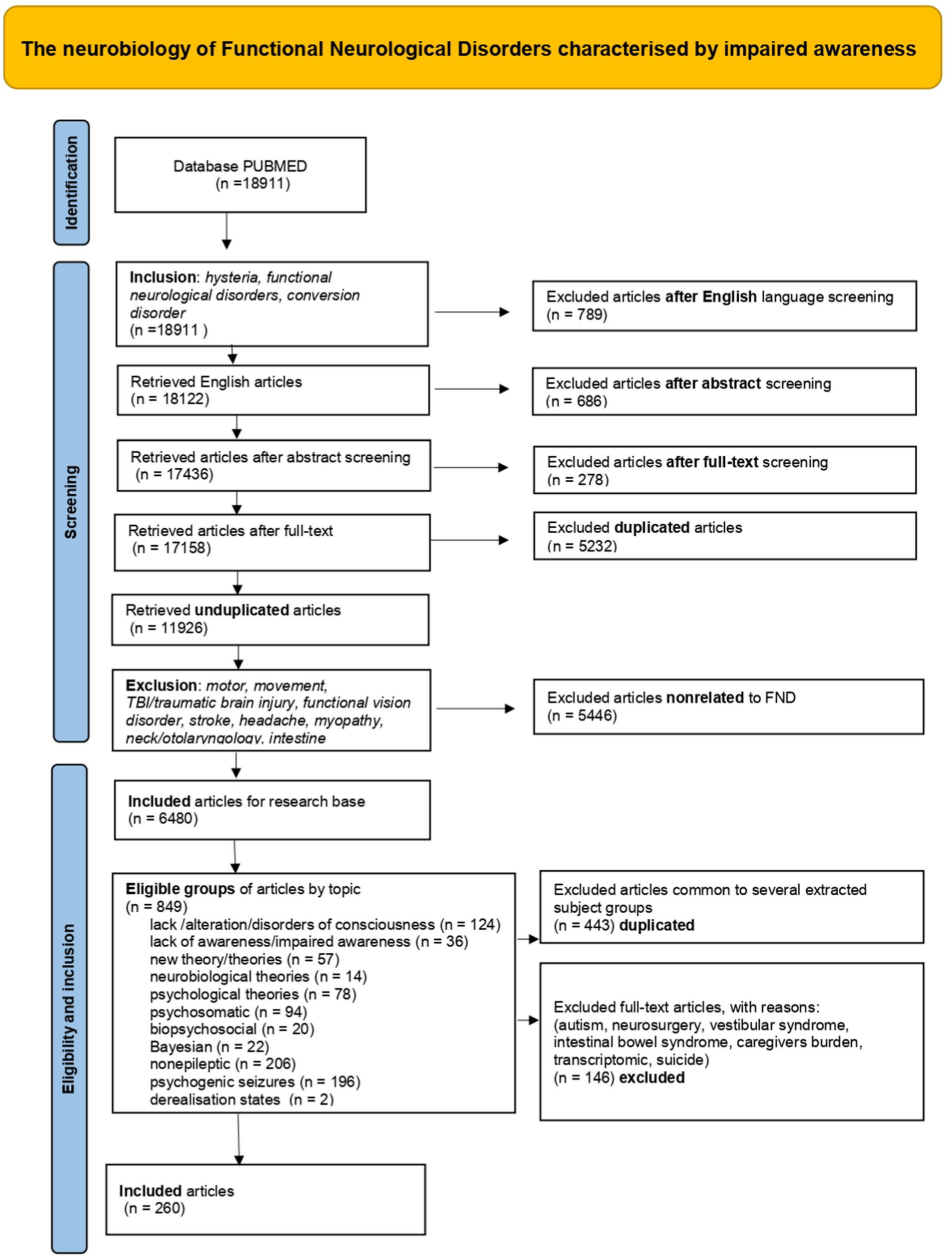
PRISMA flow diagram.

We performed a comprehensive literature search in order to ascertain neuroimaging studies, clinical reports and proposed theoretical models to contextualise the neurobiology of FIAD within social, cultural, and psychological perspectives. A PRISMA flow diagram was used to structure this search (Figure 2).

Our PUBMED search covered the years 2010–2022 with the extraction of articles according to the following selection criteria: hysteria, functional neurological disorders, and conversion disorder. This choice was made to follow the narrative of the most updated review of the field published in 2012 by Edwards et al. (64).

The search was first performed with the text string ‘hysteria’ (3,469 extracted records); a second time, the text string ‘functional neurological disorders’ was used (12,442 extracted records); a third time, the text string ‘conversion disorder’ (3,000 extracted records), for a total of 18,911 selected records.

The English language filter was introduced, and the search was repeated with the same text strings as in Step 1: once with the text string ‘hysteria’ (3,221 extracted records); a second time with the text string ‘functional neurological disorders’ (12,059 extracted records); a third time with the text string ‘conversion disorder’ (2,842 extracted records), for a total of 18,122 selected records.

The Abstract filter was introduced, and the search was repeated: first, with the text string ‘hysteria’ (2,984 extracted records); second, with the text string ‘functional neurological disorders’ (11,788 extracted records); third, with the text string ‘conversion disorder’ (2,664 extracted records), for a total of 17,436 records drawn.

The Full-text filter was selected, and the search was repeated for the topics of interest: a first time with the text string ‘hysteria’ (2,914 records extracted); a second time with the text string ‘functional neurological disorders’ (11,634 records extracted); a third time with the text string ‘conversion disorder’ (2,610 records extracted), for a total of 17,158 selected records.

We then eliminated duplicate records, i.e., records common to single-text string searches, by typing the text string ‘(hysteria) OR (functional neurological disorders) OR (conversion disorder)’. 11,926 non-duplicate records were extracted; thus, 5,232 duplicate articles were discarded.

An attempt was then made to exclude topics not relevant to the field of study: motor, movement, TBI/traumatic brain injury, functional vision disorder, stroke, headache, myopathy, neck/otolaryngology, intestine. To obtain this exclusion of articles, the following text string was used:

‘((hysteria) OR (functional neurological disorders)) OR (conversion disorder)) NOT (motor) NOT (movement) NOT (traumatic brain injury) NOT (TBI) NOT (functional vision disorder) NOT (stroke) NOT (headache) NOT (myopathy) NOT (neck) NOT (otolaryngology) NOT (intestine)’.

6,480 articles were extracted from the database, which resulted in the exclusion of 5,446 articles not related to FIAD. Our choice of excluding motor functional neurological disorders (mFND) was motivated by the aim of focusing on « impaired awareness » signs. However, some relevant papers on mFND for the purpose of our review were manually retrieved.

Of all the articles found with the selection query and filters applied, those concerning the following topics were further selected:

1. lack/alteration/disorder of consciousness (124 records).
2. lack of awareness/impaired awareness (36 records).
3. new theory/theories (57 records).
4. neurobiological theories (14 records).
5. psychological theories (78 records).
6. psychosomatic (94 records).
7. biopsychosocial (20 records).
8. Bayesian (22 records).
9. Nonepileptic (206 records).
10. psychogenic seizures (196 records).
11. derealisation states (2 records).

The 11 groups of articles were extracted by typing the following text strings into the Pubmed search field:

1. (((hysteria) OR (functional neurological disorders)) OR (conversion disorder)) NOT (motor) NOT (movement) NOT (traumatic brain injury) NOT (TBI) NOT (functional vision disorder) NOT (stroke) NOT (headache) NOT (myopathy) NOT (neck) NOT (otolaryngology) NOT (intestine) AND ((Lack consciousness) OR (alteration consciousness) OR (disorders of consciousness)).
2. (((hysteria) OR (functional neurological disorders)) OR (conversion disorder)) NOT (motor) NOT (movement) NOT (traumatic brain injury) NOT (TBI) NOT (functional vision disorder) NOT (stroke) NOT (headache) NOT (myopathy) NOT (neck) NOT (otolaryngology) NOT (intestine) AND ((lack awareness) OR (impaired awareness)).
3. (((hysteria) OR (functional neurological disorders)) OR (conversion disorder)) NOT (motor) NOT (movement) NOT (traumatic brain injury) NOT (TBI) NOT (functional vision disorder) NOT (stroke) NOT (headache) NOT (myopathy) NOT (neck) NOT (otolaryngology) NOT (intestine) AND ((new theory) and (theories)).
4. (((hysteria) OR (functional neurological disorders)) OR (conversion disorder)) NOT (motor) NOT (movement) NOT (traumatic brain injury) NOT (TBI) NOT (functional vision disorder) NOT (stroke) NOT (headache) NOT (myopathy) NOT (neck) NOT (otolaryngology) NOT (intestine) AND (neurobiological theories).
5. (((hysteria) OR (functional neurological disorders)) OR (conversion disorder)) NOT (motor) NOT (movement) NOT (traumatic brain injury) NOT (TBI) NOT (functional vision disorder) NOT (stroke) NOT (headache) NOT (myopathy) NOT (neck) NOT (otolaryngology) NOT (intestine) AND (psychological theories).
6. ((hysteria) OR (functional neurological disorders)) OR (conversion disorder)) NOT (motor) NOT (movement) NOT (traumatic brain injury) NOT (TBI) NOT (functional vision disorder) NOT (stroke) NOT (headache) NOT (myopathy) NOT (neck) NOT (otolaryngology) NOT (intestine) AND (psychosomatic).
7. (((hysteria) OR (functional neurological disorders)) OR (conversion disorder)) NOT (motor) NOT (movement) NOT (traumatic brain injury) NOT (TBI) NOT (functional vision disorder) NOT (stroke) NOT (headache) NOT (myopathy) NOT (neck) NOT (otolaryngology) NOT (intestine) AND (biopsychosocial).
8. (((hysteria) OR (functional neurological disorders)) OR (conversion disorder)) NOT (motor) NOT (movement) NOT (traumatic brain injury) NOT (TBI) NOT (functional vision disorder) NOT (stroke) NOT (headache) NOT (myopathy) NOT (neck) NOT (otolaryngology) NOT (intestine) AND (bayesian).
9. (((hysteria) OR (functional neurological disorders)) OR (conversion disorder)) NOT (motor) NOT (movement) NOT (traumatic brain injury) NOT (TBI) NOT (functional vision disorder) NOT (stroke) NOT (headache) NOT (myopathy) NOT (neck) NOT (otolaryngology) NOT (intestine) AND (nonepileptic).
10. (((hysteria) OR (functional neurological disorders)) OR (conversion disorder)) NOT (motor) NOT (movement) NOT (traumatic brain injury) NOT (TBI) NOT (functional vision disorder) NOT (stroke) NOT (headache) NOT (myopathy) NOT (neck) NOT (otolaryngology) NOT (intestine) AND (psychogenic seizures).
11. (((hysteria) OR (functional neurological disorders)) OR (conversion disorder)) NOT (motor) NOT (movement) NOT (traumatic brain injury) NOT (TBI) NOT (functional vision disorder) NOT (stroke) NOT (headache) NOT (myopathy) NOT (neck) NOT (otolaryngology) NOT (intestine) AND (derealisation states).

Duplicated articles were eliminated, i.e., 443 papers found several times in the searched topic groups.

Although eligible, other full-text articles were excluded as not pertinent, i.e., 146 papers (autism, neurosurgery, vestibular syndrome, intestinal bowel syndrome, caregiver burden, transcriptomic, suicide).

## Literature search results

A total of 260 unique articles was selected.

The selected record sets were saved in two types of files. One, in. csv format, included titles and authors’ list, whereas the other, in Pubmed format, contained the articles’ abstract.

We then generated one large. csv file, in which the individual. csv files for the searched article groups were imported and combined. An abstract column was added and abstracts from Pubmed format file were added to the unique. csv file.

The single. csv file was uploaded on Asreview, an open-source machine learning framework, that checks and helps authors’ decisions on paper inclusion for reviews (288).

Citation chaining was also performed. Both *backward* –we identified and examined references of articles from the original search – and *forward* – we researched the sources that cited original search articles to find more recent material covering the same topics (289).

The PRISMA diagram (Figure 2) summarises the structure of this systematic review, with a summary of the selected publications.

## Data availability statement

The original contributions presented in the study are included in the article/supplementary material, further inquiries can be directed to the corresponding author.

## Author contributions

BM: design, systematic literature search, selection and interpretation, methodology, visualisation, and writing. MM: conceptualization, methodology, supervision, validation, and writing. LC: conceptualization, design, supervision, validation, and writing. All authors contributed to the article and approved the submitted version.

## Funding

BM is supported by Scuola Superiore Sant’Anna, Pisa, as an honour course student. The Wellcome Trust Centre for Human Neuroimaging is funded by the Wellcome Trust. The Max Planck—University College London Centre for Computational Psychiatry and Ageing Research is a joint initiative of the Max Planck Society and UCL. MM receives support from the NIHR UCLH Biomedical Research Centre. LC is supported by the Leverhulme Doctoral Training Programme for the Ecological Study of 27 the Brain (DS-2017-026).

## Conflict of interest

The authors declare that the research was conducted in the absence of any commercial or financial relationships that could be construed as a potential conflict of interest.

## Publisher’s note

All claims expressed in this article are solely those of the authors and do not necessarily represent those of their affiliated organizations, or those of the publisher, the editors and the reviewers. Any product that may be evaluated in this article, or claim that may be made by its manufacturer, is not guaranteed or endorsed by the publisher.

## Footnotes

^1^The history of « hysteria » *per se* is often oversimplified, but outside our scope here.
